# Supplementary material for: A distinct mechanism of C-type inactivation in the Kv-like KcsA mutant E71V
Source: Nat Commun. 2022 Mar 23;13:1574. doi: 10.1038/s41467-022-28866-9 (PMC8943062; doi:10.1038/s41467-022-28866-9)
Supplement: Supplementary file 2 — Reporting Summary [file 41467_2022_28866_MOESM2_ESM.pdf]

nature portfolio

Corresponding author(s): Benoit Roux

Last updated by author(s): Jan 9, 2022

Reporting Summary

Nature Portfolio wishes to improve the reproducibility of the work that we publish. This form provides structure for consistency and transparency in reporting. For further information on Nature Portfolio policies, see our Editorial Policies and the Editorial Policy Checklist.

Statistics

For all statistical analyses, confirm that the following items are present in the figure legend, table legend, main text, or Methods section.

n/a	Confirmed	
☐	☒	The exact sample size (*n*) for each experimental group/condition, given as a discrete number and unit of measurement
☒	☐	A statement on whether measurements were taken from distinct samples or whether the same sample was measured repeatedly
☒	☐	The statistical test(s) used AND whether they are one- or two-sided<br>*Only common tests should be described solely by name; describe more complex techniques in the Methods section.*
☒	☐	A description of all covariates tested
☒	☐	A description of any assumptions or corrections, such as tests of normality and adjustment for multiple comparisons
☐	☒	A full description of the statistical parameters including central tendency (e.g. means) or other basic estimates (e.g. regression coefficient) AND variation (e.g. standard deviation) or associated estimates of uncertainty (e.g. confidence intervals)
☒	☐	For null hypothesis testing, the test statistic (e.g. *F*, *t*, *r*) with confidence intervals, effect sizes, degrees of freedom and *P* value noted<br>*Give P values as exact values whenever suitable.*
☒	☐	For Bayesian analysis, information on the choice of priors and Markov chain Monte Carlo settings
☒	☐	For hierarchical and complex designs, identification of the appropriate level for tests and full reporting of outcomes
☒	☐	Estimates of effect sizes (e.g. Cohen's *d*, Pearson's *r*), indicating how they were calculated

Our web collection on statistics for biologists contains articles on many of the points above.

Software and code

Policy information about availability of computer code

Data collection	X-ray diffraction was collected with XDS software.<br>NMR data were collected with topspin version 4.0
Data analysis	Data processing: imosflm, XDS, pointless, aimless<br>Structure determination: PhaserMR and COOT<br>Electron density map generation and visualizations: Phoenix and COOT<br>MD simulations, we used the NAMD software package (version 2.1.1)<br>X-ray Structure refinement was done with REFMAC version<br>For the annotation of NMR signals (all commercial software): Topspin 4.0, Sparky.<br>For the analysis of relaxation data (commercial software): GrapPad Prism version 7.0a for Mac OS X.

For manuscripts utilizing custom algorithms or software that are central to the research but not yet described in published literature, software must be made available to editors and reviewers. We strongly encourage code deposition in a community repository (e.g. GitHub). See the Nature Portfolio guidelines for submitting code & software for further information.

Data

Policy information about availability of data

All manuscripts must include a data availability statement. This statement should provide the following information, where applicable:

- Accession codes, unique identifiers, or web links for publicly available datasets
- A description of any restrictions on data availability
- For clinical datasets or third party data, please ensure that the statement adheres to our policy

The x-ray structures are deposited the PDB (rcsb.org) with code: 7MHR, 7MHX, 7MJT, 7MK6, 7MUB.
NMR assignments are deposited under under accession codes 51180 and 51181.

Data availability statement:
Data supporting the findings of this manuscript are available from the corresponding author on reasonable request. The solid-state NMR assignments of WT KcsA (pH 7, 100 mM K+; pH 4, 0 mM+; pH7, 5 mM Ba2+) and E71V (pH 7, 100 mM K+; pH 3, 0 mM+; pH 7, 5 mM Ba2+; pH3, 5 mM Ba2+) generated in this study have been deposited in the BMRB data base under accession codes 51180 [https://dx.doi.org/10.13018/BMR51180] and 51181 [https://dx.doi.org/10.13018/BMR51181]. The X-ray data generated in this study have been deposited in the PDB database under accession codes 7MHR [http://doi.org/10.2210/pdb7MHR/pdb], 7MHX [http://doi.org/10.2210/pdb7MHX/pdb], 7MJT [http://doi.org/10.2210/pdb7MJT/pdb], 7MK6 [http://doi.org/10.2210/pdb7MK6/pdb], and 7MUB [http://doi.org/10.2210/pdb7MUB/pdb]. The data underlying Figs. 3d, 3e, 5h, 6a, and 6d are provided as a Source Data file.

Field-specific reporting

Please select the one below that is the best fit for your research. If you are not sure, read the appropriate sections before making your selection.

☒ Life sciences ☐ Behavioural & social sciences ☐ Ecological, evolutionary & environmental sciences

For a reference copy of the document with all sections, see nature.com/documents/nr-reporting-summary-flat.pdf

Life sciences study design

All studies must disclose on these points even when the disclosure is negative.

Sample size	No statistical methods were used to predetermine sample size. The data for x-ray crystallography studies were determined using single crystals. Data reporting the number of reflections used in structure determination and refined are reported in supplementary Table 1.
Data exclusions	No dataset was excluded from the structural analysis
Replication	Structural studies were performed using single crystals diffraction.
Randomization	In x-ray diffraction 5% of the data was randomly chosen to calculate Free R factor, shown in supplementary table 1.
Blinding	Blinding not applicable as no grouping were performed in this study.

Reporting for specific materials, systems and methods

We require information from authors about some types of materials, experimental systems and methods used in many studies. Here, indicate whether each material, system or method listed is relevant to your study. If you are not sure if a list item applies to your research, read the appropriate section before selecting a response.

Materials & experimental systems

n/a	Involved in the study	
☐	☒	Antibodies
☒	☐	Eukaryotic cell lines
☒	☐	Palaeontology and archaeology
☒	☐	Animals and other organisms
☒	☐	Human research participants
☒	☐	Clinical data
☒	☐	Dual use research of concern

Methods

n/a	Involved in the study	
☒	☐	ChIP-seq
☒	☐	Flow cytometry
☒	☐	MRI-based neuroimaging

Antibodies

Antibodies used	Fab fragment were used for crystallization purpose. Recombinantly expressed and purified in house. The sequence and structure of the Fab is deposited in the pdb with accession code: 1K4C, identifier: >1K4C_2|Chain A/B|antibody Fab fragment light chain|Mus

musculus (10090)

Validation	Fab fragemtn obtained form mouse antibodies. Full description is available in the pdb: "https://www.rcsb.org/fasta/entry/1K4C/display". 1K4C, identifier: >1K4C_2|Chain A/B|antibody Fab fragment light chain|Mus musculus (10090)
